# Education and Training of Emergency Medical Teams: Recommendations for a Global Operational Learning Framework

**DOI:** 10.1371/currents.dis.292033689209611ad5e4a7a3e61520d0

**Published:** 2016-10-21

**Authors:** Nieves Amat Camacho, Amy Hughes, Frederick M. Burkle, Pier Luigi Ingrassia, Luca Ragazzoni, Anthony Redmond, Ian Norton, Johan von Schreeb

**Affiliations:** Centre for Research on Health Care in DisastersKarolinska Institutet; Humanitarian and Conflict Response Institute, University of Manchester, Manchester, UK; Harvard Humanitarian Initiative, Harvard University, Cambridge, Massachusetts, USA; Research Center in Emergency and Disaster Medicine and Computer Science applied to Medicine (CRIMEDIM); Università del Piemonte Orientale, Novara, Italy; CRIMEDIM - Research Center in Emergency and Disaster Medicine and Computer Science applied to Medical Practice; Università del Piemonte Orientale, Novara, Italy; Humanitarian and Conflict Response Institute, University of Manchester, Manchester, UK; Emergency Medical Teams (EMT) Project – Policy, Practice and Evaluation Unit, Emergency Risk Management and Humanitarian Response, World Health Organization, Geneva, Switzerland; Centre for Research on Health Care in Disasters, Health System and Policy, Department of Public Health Sciences, Karolinska Institutet, Stockholm, Sweden

## Abstract

An increasing number of international emergency medical teams are deployed to assist disaster-affected populations worldwide. Since Haiti earthquake those teams have been criticised for ill adapted care, lack of preparedness in addition to not coordinating with the affected country healthcare system. The Emergency Medical Teams (EMTs) initiative, as part of the Word Health Organization’s Global Health Emergency Workforce program, aims to address these shortcomings by improved EMT coordination, and mechanisms to ensure quality and accountability of national and international EMTs. An essential component to reach this goal is appropriate education and training. Multiple disaster education and training programs are available. However, most are centred on individuals’ professional development rather than on the EMTs operational performance. Moreover, no common overarching or standardised training frameworks exist. In this report, an expert panel review and discuss the current approaches to disaster education and training and propose a three-step operational learning framework that could be used for EMTs globally. The proposed framework includes the following steps: 1) ensure professional competence and license to practice, 2) support adaptation of technical and non-technical professional capacities into the low-resource and emergency context and 3) prepare for an effective team performance in the field. A combination of training methodologies is also recommended, including individual theory based education, immersive simulations and team training. Agreed curriculum and open access training materials for EMTs need to be further developed, ideally through collaborative efforts between WHO, operational EMT organizations, universities, professional bodies and training agencies.  Keywords: disasters; education; emergencies; global health; learning

## Introduction

Disasters regularly have devastating effects on populations worldwide 1 ^,^ 2. To assist affected countries an increasing number of international emergency medical teams has been deployed 3. Concerns regarding the standard of medical care provided and the lack of preparedness of the teams have been raised. Health practitioners have been observed to work outside their scope of practice and license 4 ^,^ 5, and teams have lacked the basic capacities and means to be fully self-sufficient 2 ^,^ 6. Additional concerns have been highlighted regarding the lack of cultural awareness and coordination with local authorities as well as international agencies 7 ^,^ 8 ^,^ 9. More recently, the response to the West African Ebola epidemic has shown critical gaps in the timeliness, coordination and effectiveness of international emergency medical teams responding to outbreaks 10.

The ‘Foreign Medical Teams’ (FMTs) initiative evolved in 2010 under the umbrella of the World Health Organisation (WHO), the Global Health Cluster and other actors, with the aim to improve the quality and accountability of international emergency medical teams responding to disasters. In 2013, the FMT Working Group published a first edition of the ‘Classification and minimum standards for Foreign Medical Teams in sudden onset disasters’, in which capacities, services and minimum deployment standards for FMTs were defined 11. A global list of quality assured and classified FMT organizations was launched in July 2015. A change of name from FMT to Emergency Medical Teams (EMT) with a pre-fix to differentiate International and National teams (I-EMT and N-EMT) was endorsed at the global meeting held in Panama in December 2015. This was in recognition of the importance of national and international teams working collaboratively to maximise the response to large scale health emergencies (Table 1).

**Figure:**
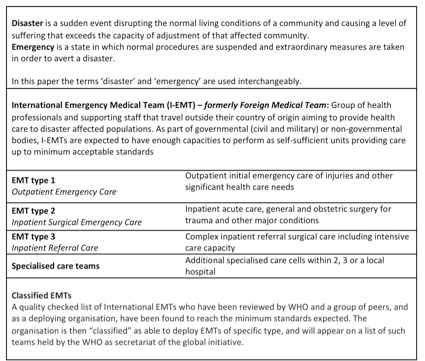
**Table 1.** EMT and disaster related definitions

The World Health Assembly 2015 recognised the need for a global health surge capacity and the establishment of the Global Health Emergency Workforce (GHEW), of which the EMT initiative is a part. The GHEW aims to improve coordination, readiness and quality assurance in the deployment of EMTs and individual experts such as those deployed through the Global Outbreak Alert and response Network (GOARN) and other networks and partnerships 12.

To improve the quality and professionalism of deployed teams, a coherent approach to education and training has been identified as a key next step 8. A standardised learning framework is needed to assist EMTs to prepare for response and allow quality assurance mechanisms for the EMT initiative. Organisations wishing to be EMT classified will be required to reveal their training strategies.

Multiple organisations and universities have developed education and training programmes for disaster and emergency response; with a significant variation in scope, curriculum and quality 13 ^,^ 14 ^,^ 15 ^,^ 16. The lack of common standards to guide education and training design and provision have been highlighted 13 ^,^ 17 ^,^ 18. In addition, many of the proposed training models are focused on individuals, rather than multidisciplinary EMTs 19. The so called ‘competency-based models’ have been recommended as the basis for education and training in the disaster field by several authors 20 ^,^ 21 ^,^ 22 ^,^ 23 ^,^ 24 ^,^ 25 ^,^ 26 ^,^ 27 ^,^ 28. Such models are promoted as a way to standardise the training of individuals and contribute to the professionalisation of the discipline, but reviews of available competency models have shown limitations in their practical application 18 ^,^ 29. Although some authors have suggested possible ways to facilitate their alignment to practice 30 ^,^ 31, no competency models have led to a systematic and operationally focused framework to guide EMT organisations through an agreed training pathway for their teams.

The aim of this study is to explore and reflect on current practices related to disaster education and training and suggest key components for an operational EMT learning framework. This targets primarily I- EMTs.

## Methods

This work has evolved out of the EMT process in which the authors are involved at different levels. The authors hold extensive experience in both medical field work in disaster contexts and disaster training development and implementation. Based on this, a first group (NAC, AH, IN, JvS) was formed to develop an EMT operational training framework that would contribute to quality and accountability mechanisms within the EMT initiative. A literature review was done compiling available literature from trainings and educational frameworks within the field of disaster medicine. Published literature search was performed using PubMed, EMBASE and Google Scholar. Since a limited amount of references about operational training was found, a search for grey literature followed. That included information from internet sites and other information made available for the authors by EMT organizations. The results were categorized and discussed by NAC, AH, IN and JvS, and a first draft with training recommendations was presented to the expert panel consisting of the remaining authors. Following sets of 5 revisions a final Global Operational Learning Framework was defined. The results presented are based on the discussion process that lead up to the framework.

## Results

**Current education and training for disaster and emergency response**

Mainly individual education and training options are available to help prepare professionals engaging in disaster response. Individuals can strengthen existing professional skills and develop technical and context specific capacities through Masters level studies or short courses delivered by universities, training agencies or EMT organisations themselves, many of those trainings being recently compiled by Jacquet et al. 13. Two papers also gather a comprehensive compilation of postgraduate education programmes related to disasters offered in North America and Europe 14 ^,^ 15. Delivered online or face-to face, the numerous available courses cover multiple subjects; as broad as Global Health or as specific as nutrition or logistics in low-resource settings. The previously mentioned competency based models, mostly compiled in two systematic reviews 18 ^,^ 29, aimed to guide standardized curriculum design but their application in practical courses have not yet been documented. Training modalities also vary, from theory-based lectures and discussions, to case-scenario exercises and simulations 14 ^,^ 15.

Although it is acknowledged that EMT deploying organizations provide team training little evidence of their practices is available. The training approach followed by strong and experienced EMTs and organisations involved in emergency response have hardly been studied, even if many lessons may be learnt from their experiences. These organizations comprise emergency teams from international organizations, governments or well-known NGOs, as well as police, Fire and Rescue, ambulance services or militaries. They often follow an operational approach to training, immersing their teams into contexts they will likely be exposed to once in the field. Simulations, teamwork, pre-deployment preparation and the inclusion of regional and national actors are key features of their training practices 32 ^,^ 33. Table 2 illustrates some examples of these practices, which should be especially considered when designing an operational approach to training for EMTs.

**Figure:**
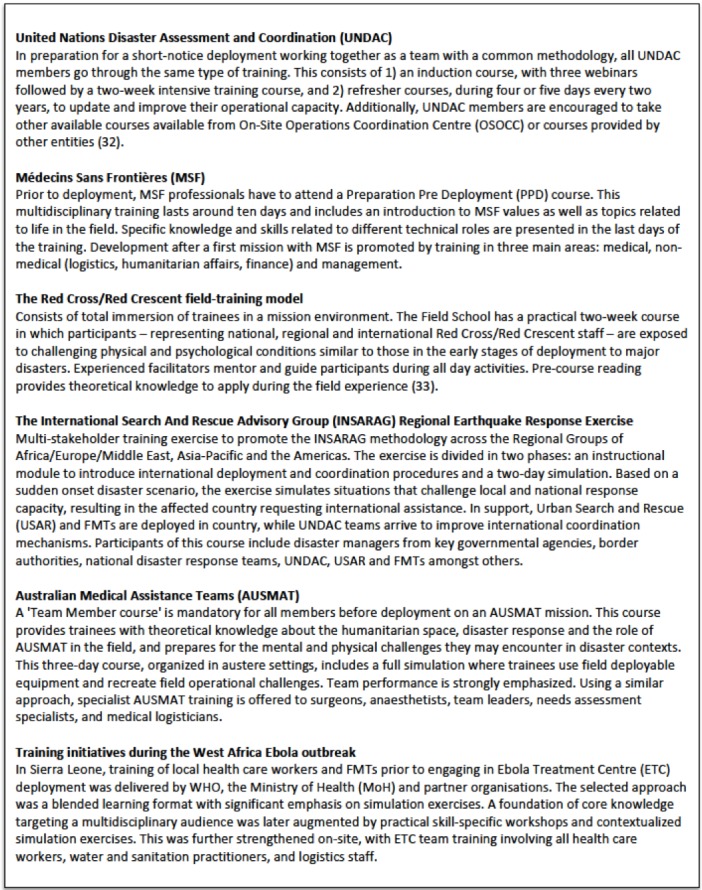
**Table 2.** Examples of emergency training by relevant EMTs and emergency organizations

**Recommendations for a global operational learning framework for EMTs**

After a critical analysis and discussion around EMT education and training current practice and needs a systematic approach linked to current WHO EMT standards is presented. It recognises both individual competencies and team dynamics within the procedures of a field deployable agency as being equally important factors for an effective response.

***1. Three-step learning process***

The three steps proposed below (Figure 1) are designed to:

1. Ensure professional competence and license to practice
2. Support adaptation of technical and non-technical professional capacities into low-resource and emergency context
3. Prepare for an effective team performance in the field

**Three-step learning process for EMTs:**
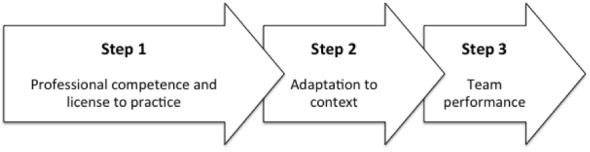

With different formats and levels of complexity, several authors have already mentioned comparable stages of competency, training and development 20 ^,^ 21 ^,^ 25 ^,^ 31. The classification suggested in this paper simplifies the current approaches and provides a clear picture of the minimum team capacities needed to deploy as an EMT, leaving space for the future design of pathways for those seeking sector professional development. As the three steps presented aim to be the minimum standard for education and training, no individuals should be deployed to the field without going through all steps; each being considered equally relevant for EMT performance during disasters. It is the responsibility of EMT organisations to ensure that staff has gone through all three steps. Well-trained EMTs will result in more effective performance and better care for populations affected, rather than just the career development of an individual.

Step 1 – Professional competence and license to practice

The core standards recommended within the 2013 publication ‘Classification and minimum standards for Foreign Medical Teams in sudden onset disasters’ 11 , already 7 underline the first step to consider in relation to EMT education and training: ‘FMTs must ensure all their staff are registered to practice in their home country and have licence for the work they are assigned to do during their deployment, as well as showing expertise in their field of practice’.

Although EMT organisations are not in charge of providing this education, they have the responsibility to ensure their staff have been trained and accredited by a competent authority for their field of health practice. This learning step must occur before a professional becomes part of the EMT. For example, an EMT member would comply with step 1 by getting a medical degree, a license to practice from their specific professional body and relevant working experience in his/her home country.

Step 2 – Adaptation to context

Another established core standard for EMTs 11 is the need to ensure members are appropriately trained for the context in which they will work. Step 2 of the recommended learning approach emphasises that EMT members must adapt exiting professional skills and competencies to the resource limited emergency contexts.

Step 2 training should be done well ahead of deployment. Courses and education platforms should besides professional context adaptation focus on developing skills to critically assess and analyse the situation in order to ensure that priority is given to the most essential health needs of the population and a capacity to triage, based on public health priorities and available resources. Examples of technical training courses are those providing context specific clinical skills (surgery, wound care, paediatrics, mass casualty), public health (disease prevention, health systems, management of epidemics) or logistics (shelter, water and sanitation). Examples of non-technical subjects include ethics, cultural awareness, leadership, communication or understanding of the humanitarian structure.

EMT organisations may have the capacity to facilitate this learning step internally, but can also use external partners, such as universities or training companies, to organise and deliver it to their EMT members. To receive step 2 education and training, 8 individuals could also enrol in existing university based Masters programmes or short courses related to disaster and emergency management or health in disasters.

Step 3 – Team performance

Previous professional expertise and the completion of emergency and low-resource adapted individual courses provide the basis for good practice in the field, but do not ensure the successful performance of a team deployed into a disaster 19.

EMT members need to prepare for their deployment as part of a multidisciplinary team integrated within an EMT deploying organisation. This training goes beyond the individually focused training of step 1 and 2 above, and puts teamwork and EMTs’ specific procedures into focus. All EMTs should offer pre-deployment courses in order to transfer to its members the values and mandate of the organisation, its main protocols, communication pathways, security guidelines, teamwork dynamics, basic aspects of personal health and travel, and other subjects related to deployment working and living conditions. This training must be practical and multidisciplinary, and inclusive of all health care workers and non-medical professions. It is anticipated that the team members who train together may not always be the same that deploy together; but a standardised pre-deployment course should allow those taken from an organisation’s roster to work as an effective team. Step 3 training is the responsibility of the EMT agency but may be delivered in partnership with other training providers.

***2. Considerations for training delivery***

*From theory to practice*

We assume both theoretical and practical learning to be a base for any professional field. In the initial phase, theoretical education facilitates knowledge acquisition, but soon trainees require more practical and hands-on training sessions in which to apply these theories. Following the same pattern, EMT learning process moves from theory to practice as deployment comes closer. Face-to-face or e-learning theoretical courses should then be followed by real or simulation based, practical training. Although it will be best to expose trainees to the real context of the disaster and emergency in which 9 they will ultimately be working, this is rarely possible. As an alternative, simulation based training can offer a feasible and effective approximation to real-life practice in the field 34 ^,^ 35 ^,^ 36.

The traditional real-life drills and table top exercises can be difficult to organise due to the length of time and amount of resources required for design, execution and review. Technologically based approaches to disaster training appear promising in their ability to bridge the gaps between other common training formats. For example, during the recent Ebola outbreak emergency, virtual reality (VR) training was designed by replicating an Ebola Treatment Centre (ETC) to create a safe and realistic environment in which trainees could gain realistic skills. Although it had some limitations, a VR training programme produced a cost-effective option and increased access to simulation training 37.

Although more costly, exposure of trainees to low-resource settings could be achieved through the establishment of field training facilities, similar to the Red Cross/ Red Crescent Field School included in table 2. Agreements between academic institutions and non-governmental organisations (NGOs) can offer similar opportunities. An example of this was a project in which online training was combined with a medical apprenticeship in low-income countries during an anaesthesia and intensive care medicine residency in Italy 38.

*From individual to team training*

Although the pursuit of individual expertise is important, the scale of disaster operations requires an organised response by teams of interdependent members, who can incorporate individual efforts into coordinated actions 39. Thus, understanding the roles of other professional groups included in the team and learning how to work together to reach a common goal are key aspects of a successful disaster response 40. Moreover, team training across different clinical contexts has proven to impact positively upon healthcare teamwork processes and it has been associated with improvements in patient outcomes 41 ^,^ 42. The team approach is also valid for the whole system responding to the disaster – i.e. working as a multidisciplinary and interdisciplinary team with national staff and other organisations.

While EMT training is mandatory it cannot replace experiential learning and field mentorship. The team composition should be considered carefully, with a balance between senior and junior staff that allows a quality performance of the team and the mentorship of its junior members. This is likely to be inappropriate in the first response team, but to be encouraged in second and subsequent staff rotations as a situation stabilises. This important recommendation is also recognised in the minimum standards for FMTs 11.

*Just-in-time training*

Just-in-time training (JITT) is recognised in medical education as a valuable and effective training method to disseminate newer concepts or seldom-performed procedures 43 ^,^ 44. Already suggested as relevant to EMT training 30, potential JITT modules will introduce additional skills and knowledge to the staff just before deploying into specific contexts – e.g. clinical management of Ebola patients or description of trauma national protocols of a disaster-affected country. JITT courses – short and well defined in their scope – could also be organised to present updates of former EMT guidelines and procedures, or refresh important concepts after a period without deploying to the field or since original pre/deployment training.

*Skills mix and team composition*

Training matrices can be used to identify which team members require which skills and to what depth of knowledge. The idea of team rather than individual skills development is important. A surgical team in a Type 2 facility for example must be able to perform emergency general surgical procedures such as a laparotomy for trauma, as well as wound and limb injury care that has orthopaedic and plastic surgical elements, and be able to manage an emergency caesarean section. In that case the organisation must decide to either bring a surgical team encompassing the different specialised surgeons or to bring generalist surgeons who have specific skills in each of these areas. Similarly all team members must be aware of safety and security procedures, but at least one person from the team should have in-depth knowledge of this area to support team safety and security planning and operations.

*Need to complete all levels of training*

Individuals or groups who were not self-sufficient in the field contributed to the chaos in recent disasters and added an inappropriate burden to the affected country without contributing to the care of the affected population. Academic institutions providing Step 2 education and training must guide their graduates on the appropriate mechanisms to deploy. If not there is a risk of encouraging more spontaneous and unsupported responders, in opposition to a systematic approach to emergency response led by wellprepared teams.

Recent EMT field deployments have exposed a lack of public health skills amongst some deployed clinical specialists, for instance during the Ebola outbreak, when public health understanding was especially relevant 45. This again reflects the need for a multidimensional learning framework in which contextual adaptation is crucial.

## Next steps

The framework proposed in this paper – backed-up by international experts in the subject – encompasses existing successful initiatives where these principles have already been applied and documented in relation to EMT training, and complies with recognised WHO EMT standards. Although we believe this is a strong base for a global framework, its endorsement and testing by other EMT organizations should follow this publication.

This paper suggests the key components for an operational learning framework for EMTs but recommended curriculum content for each of its steps needs to be further defined and agreed. This should come with strong input from EMT organisations, rather than be driven solely by academic institutions. This could occur as a dedicated working group under the auspices of the WHO EMT initiative. The EMT WHO website could be used to share current education and training programmes by well-known organisations that could serve as guidance for other EMTs. Further development of open access training materials available to national and international EMTs will contribute to the quality improvement of the training practices.

Evaluation and accreditation of courses and participants remains an important area for discussion. The completion of exercises by trainees during a classroom course or a simulation exercise does not ensure they are competent to perform appropriately once in the field. Effective mechanisms to assess trainees´ capacities should be established. For example, training-participants’ performance during simulation exercises can be assessed through debriefing sessions, led by specialised facilitators who can challenge inadequate 12 practices identified and recommend improvements. Supervision and assessments during and after deployments can also contribute to the evaluation of the level of competence of EMT professionals once they are working within an organisation. Tools to allow meaningful and constructive debriefing and feedback from disaster affected countries also need to be developed. The establishment of a minimum dataset and uniform reporting, including collaborative and coordinated post-deployment research and evaluation, will strengthen the development of the learning framework.

## Conclusions

Multiple attempts to standardise the education and training of disaster and emergency responders have been made; these focused mainly on an individual’s professional development rather than improved team operational performance. No agreed overarching framework currently guides EMTs through the principles of training or recommends suitable training methodologies. Since a systematic approach is needed, this report suggests a three-step operational learning framework for EMTs that could be implemented by EMT organizations globally. In addition, the importance of the training modalities used is highlighted; including individual and theory based education but emphasising team and practical simulations as crucial to the operational nature of an EMT’s work. Further work is required to fully develop an agreed curriculum and open access training materials for EMTs. These training materials will also contribute to the development of N-EMTs, some of which may be offered to neighbouring countries as IEMTs. WHO, EMT organisations, universities, professional bodies and training agencies can all contribute to the development of professional and highly functioning teams, but should recognise that only a collective approach will improve EMT field performance and crucially, result in better care for the victims of large scale health emergencies.

## Competing Interests

The authors have declared that no competing interests exist.

## Data Availability Statement

All relevant data are within the paper.

## Corresponding Author

Ian Norton

Contact e-mail: nortoni@who.int
